# Synthesis and characterization of an organic–inorganic hybrid crystal: 2[Co(en)_3_](V_4_O_13_)·4H_2_O

**DOI:** 10.1107/S2052520624007509

**Published:** 2024-09-03

**Authors:** Emilie Skytte Vosegaard, Mohammad Aref Hasen Mamakhel, Vijay Singh Parmar, Andreas Dueholm Bertelsen, Bo Brummerstedt Iversen

**Affiliations:** a Department of Chemistry Aarhus University Langelandsgade 140 8000Aarhus Denmark; Polish Academy of Sciences, Poland

**Keywords:** hybrid crystal, crystal structure, electronic and magnetic properties, organic cobalt vanadium oxide

## Abstract

Organic–inorganic hybrid crystals have diverse functionalities, for example in energy storage and luminescence, due to their versatile structures. The synthesis and structural characterization of a new cobalt–vanadium-containing compound, 2[Co(en)_3_]^3+^(V_4_O_13_)^6−^·4H_2_O (**1**) is presented. The crystal structure of **1**, consisting of [Co(en)_3_]^3+^ complexes and chains of corner-sharing (VO_4_) tetrahedra, was solved by single-crystal X-ray diffraction in the centrosymmetric space group *P*1. Phase purity of the bulk material was confirmed by infrared spectroscopy, scanning electron microscopy, elemental analysis and powder X-ray diffraction. The volume expansion of **1** was found to be close to 1% in the reported temperature range from 100 to 300 K, with a volume thermal expansion coefficient of 56 (2) × 10^−6^ K^−1^. The electronic band gap of **1** is 2.30 (1) eV, and magnetic susceptibility measurements showed that the compound exhibits a weak paramagnetic response down to 1.8 K, probably due to minor Co^II^ impurities (<1%) on the Co^III^ site.

## Abstract 2

The synthesis, single-crystal structure and property characterization of a new mixed metal organic–inorganic hybrid crystal containing cobalt and vanadium, specifically 2[Co(en)_3_](V_4_O_13_)·4H_2_O, are presented.

## 1. Introduction

Organic–inorganic hybrid crystals have been studied due to their diverse properties, which are useful in a range of applications such as energy storage (Zhao *et al.*, 2023 ▸; Li *et al.*, 2020 ▸; Lu *et al.*, 2020 ▸), photo-/electro-catalysis (Lan *et al.*, 2023 ▸; Li *et al.*, 2018 ▸) and luminescence (Wang *et al.*, 2022 ▸). Much akin to the famous metal–organic frameworks, the advantageous properties arise from the very large variations in available structures with just simple modifications to the synthesis (Yao *et al.*, 2010 ▸). Usually organic–inorganic hybrid crystals are composite materials of ionic species with the cation being a metal–organic complex balancing the charge of the anionic inorganic extended structure (Hagrman *et al.*, 2001 ▸). The ‘organic’ species, typically a transition metal complex containing organic ligands, has the ability to work as a templating agent, governing morphology, structure and properties for the inorganic framework, making the versatile hybrid crystals a playground for crystal engineering and design. In the literature, the inorganic framework has shown a diverse nature from isolated simple tetrahedra of metal chalcogenides (Aschwanden *et al.*, 1993 ▸) to intricate polymetal complexes (Guo *et al.*, 2016 ▸; Yi *et al.*, 2010 ▸; Wendt *et al.*, 2016 ▸; Lan *et al.*, 2023 ▸), over extended 1-D chains (Wang *et al.*, 2004 ▸; Khan *et al.*, 2005 ▸; Liu *et al.*, 2000 ▸; Lin *et al.*, 2003 ▸) and 2-D planes (Shi *et al.*, 1999 ▸; Lu *et al.*, 2004 ▸; Zhang *et al.*, 1996 ▸), to comprehensive 3-D networks (Zhao *et al.*, 2023 ▸).

**Figure 1:**
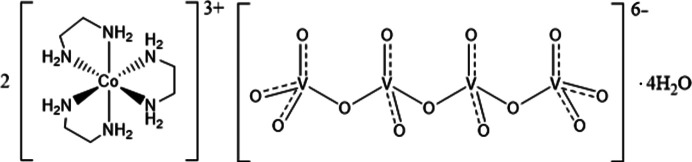
Schematic drawing of the structure of 2[Co^III^(en)_3_](V^V^_4_O_13_)·4H_2_O (**1**) with balanced charges. Both the δ and λ ;enantiomers of [Co(en)3]3+ are present in the structure.

In this study we report the hydro­thermal synthesis and structural characterization of a new cobalt–vanadium- containing compound, cobalt ethyl­enedi­amine­(en) vanadium oxide 2[Co(en)_3_]^3+^(V_4_O_13_)^6−^·4H_2_O (**1**) (Fig. 1 ▸). Cobalt and vanadium compounds have recently become the centre of attention in the search for transition-metal-containing materials with catalytic properties to replace the current rare earth metal-containing catalysts (Liardet & Hu, 2018 ▸). The reported compound **1** may be used as a precursor material for both catalytic cobalt vanadium oxides and metallic cobalt vanadium alloys.

Other compounds containing the [Co(en)_3_]^3+^ complex have mainly been studied due to the templating properties of the chiral coordination complex, making it possible to obtain stereo-selective products (Ghosh *et al.*, 2017 ▸). Only a very few mixed crystals of [Co(en)_3_]^3+^ and vanadium oxide have been reported. Aschwanden *et al.* (1993 ▸) reported the first cobalt–ethyl­enedi­amine–vanadate compound with the chemical formula [Co(en)_3_]·2V_2_O_4_·HVO_4_·6H_2_O crystallizing in the triclinic space group *P*1. Wang *et al.* (2004 ▸) and Khan *et al.* (2005 ▸) have reported a compound with the chemical formula [Co(en)_3_][V_3_O_9_]·H_2_O similar to **1**, which crystallizes in the chiral orthorhombic space group *P*2_1_2_1_2_1_ and has been proposed to act as an optically active complex. Similar 1-D and 2-D polyoxovanadate compounds with organic templates such as Co(en)_2_ (Zhang *et al.*, 1996 ▸), Co(dien)_2_ (Lin *et al.*, 2003 ▸), Co(phen)_3_ (Lu *et al.*, 2004 ▸), Ni(en)_3_ (Liu *et al.*, 2000 ▸) *etc*. are found in the literature. As for the inorganic framework, a large number of chemically similar structures of metal/pnictogen chalcogenides (Zhou & An, 2011 ▸; Guo *et al.*, 2016 ▸) have been reported, *e.g.* arsenic sulfide (Tang *et al.*, 2013 ▸) or phosphate (Wang *et al.*, 2004 ▸) with an (*n*+10) relation to the vanadium oxide reported in this study. To our knowledge, **1** has never been reported before. The product is crystalline and it has been characterized by elemental analysis, scanning electron microscopy (SEM), infrared spectroscopy (IR), as well as powder and single crystal X-ray diffraction (PXRD/SCXRD). Furthermore electronic and magnetic properties were probed by UBV–vis spectroscopy and magnetic measurements.

## 2. Experimental

### 2.1. Synthesis

Powders of the precursor materials, CoCl_2_·6H_2_O and NH_4_VO_3_, were used with molar ratios of 1:3, 1:2, 1:1 and 2:1 with a total weight of approx. 0.45 g. Each of the four mixtures were processed similarly and yielded the same product (see supporting information). Each mixture was added to glycolic acid (3 ml) and demineralized water (4 ml) in a glass tube and magnetically stirred for 1 h at room temperature. Then, ethyl­enedi­amine (en) C_2_H_4_(NH_2_)_2_ (0.8 ml) was added to this solution, and the glass tube was placed in an autoclave. The autoclave was heated at a rate of 5°C min^−1^ up to 90°C and held at this temperature for five days and then cooled to room temperature. The content of the autoclave was centrifuged and the liquid was kept at room temperature for four days to obtain golden single crystals of **1** (Fig. S1). The yield of product was 47% based on NH_4_VO_3_. During the reaction Co undergoes oxidation from Co^II^ in the precursor to Co^III^ in the final product. Byproducts of the synthesis should account for the unbalanced charges between precursor and product.

### 2.2. Single-crystal X-ray diffraction

Single-crystals of **1** were used for measurements on a Rigaku XtalLAB Synergy-S diffractometer with a monochromatic Mo *K*α microfocus sealed tube source and a HyPix-Arc 100° detector. The sample-to-detector distance was set to the minimum distance of approx. 40 mm with a scan width of 0.5 degrees per frame. Data integration and reduction were carried out using *CrysAlisPro* (Rigaku Oxford Diffraction, 2019 ▸). No outlier rejection was applied, but Friedel pairs were assumed to be equivalent, after the initial space group solution suggested 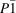. Automated empirical correction and numerical absorption correction based on a spherical model of the crystal was applied, as well as automatic error models and filters.

The crystal structure was solved and refined using an independent atom model (IAM) in *Olex2* (Dolomanov *et al.*, 2009 ▸) with *SHELXTL* (Sheldrick, 2008 ▸, 2015*a* ▸,*b* ▸). All non-hydrogen atoms were refined freely with anisotropic atomic displacement parameters (ADPs). Hydrogen atoms in the ethyl­enedi­amine ligands were refined as riding atoms with AFIX 23, meaning that they follow the position of the nearest carbon or nitro­gen, with idealized CH_2_ geometry, *e.g.* bond lengths and bond angles, and isotropic ADPs set to 120% of the neighbouring atom. Hydrogen in the water molecules were set as riding on the oxygen atom, but with free angular rotation, and the isotropic ADP set to 150% of the *U*_eq_ of the oxygen atom. Sample information, crystal sizes, unit-cell parameters and other crystallographic information, as well as quality parameters for the integration and structure solution can be seen in Table 1 ▸. All structural figures in the paper were produced using *Olex2*.

### 2.3. Methods

#### 2.3.1. Scanning electron microscopy and elemental analysis

The measurements were performed using a TESCAN CLARA as an ultra-high-resolution scanning electron microscope (SEM) equipped with an ULTIM MAX Oxford energy-dispersive X-ray microanalysis (EDX) system. The microscope was operated at high vacuum using 20 K eV energy, 3 nA and Everhart–Thornley detector.

#### 2.3.2. Fourier transform infrared spectroscopy

FT–IR spectra of **1** were recorded on a NICOLET 380 with smart orbit spectro-photometer in the 4000–400 cm^−1^ range.

#### 2.3.3. Powder X-ray diffraction

PXRD data of **1** were collected at four temperatures, 300 K, 150 K, 125 K and 107 K, using the OHGI (Kato *et al.*, 2019 ▸) detector at the RIKEN Materials Science beamline BL44B2 (Kato *et al.*, 2010 ▸; Kato & Tanaka, 2016 ▸) at SPring-8, Japan. The incident X-rays had an energy of 27.55 keV (λ = 0.45 Å) and the detectable energy threshold was set to 13.8 keV. The sample was packed into a glass capillary with an inner diameter of 0.2 mm. The dimensions of the incident beam were fixed by a collimator to 3 mm in the horizontal direction and 0.5 mm in the vertical direction. The total data-collection time at each temperature was 4 min. The obtained PXRD patterns were modelled by Rietveld refinement in *TOPAS* using the unit cell obtained from SCXRD, with refined parameters being unit cell, scale factor, profile parameters and ADPs fixed at the average value for each atomic type from the single-crystal refinement at the corresponding temperature. The H atoms were omitted from the modelling. The background was modelled using the PXRD pattern of an empty glass capillary, along with a 5th degree Chebyshev polynomial and a broad Gaussian function centred at 6.4° 2θ. Instrumental zero shift and profile parameters were obtained from a Si standard (NIST SRM640d).

#### 2.3.4. UV–vis spectroscopy

Optical diffuse reflectance measurements were performed at room temperature using a Shimadzu UV-3600 spectrometer. BaSO_4_ was used as reference sample and the spectra were recorded in the range of 200–700 nm. The band gap was estimated using a Tauc plot assuming direct allowed transition (plot of [*F*(*R*)·*h*ν]^2^ against *h*ν, where *F*(*R*) is Kubelka–Munk function and *h*ν is incident photon energy).

#### 2.3.5. Magnetic measurements

Magnetic measurements were performed using 11 mg of powdered sample loaded in a plastic sample holder and fastened to a quartz holder using Kapton tape, on a Quantum Design MPMS3 SQUID 9.7 under VSM mode. Pre-measured magnetic background correction factor due to sample holder (−2.6 × 10^−6^ emu at 500 Oe) was applied to the raw data. Diamagnetic correction of −0.5 × *M*_w_ × 10^−6^ was applied to all the molar susceptibility data. Zero field cooled (ZFC) and field cooled (FC) measurements were carried out at 500 Oe; the cooling rate was set at 2 K min^−1^. Magnetic hysteresis were performed from −7 to 7 T external field at 1.8 K with an average field sweep rate of 50 Oe s^−1^. No background correction but only diamagnetic correction was applied to the hysteresis data.

## 3. Results

### 3.1. Structure

**Figure 2:**
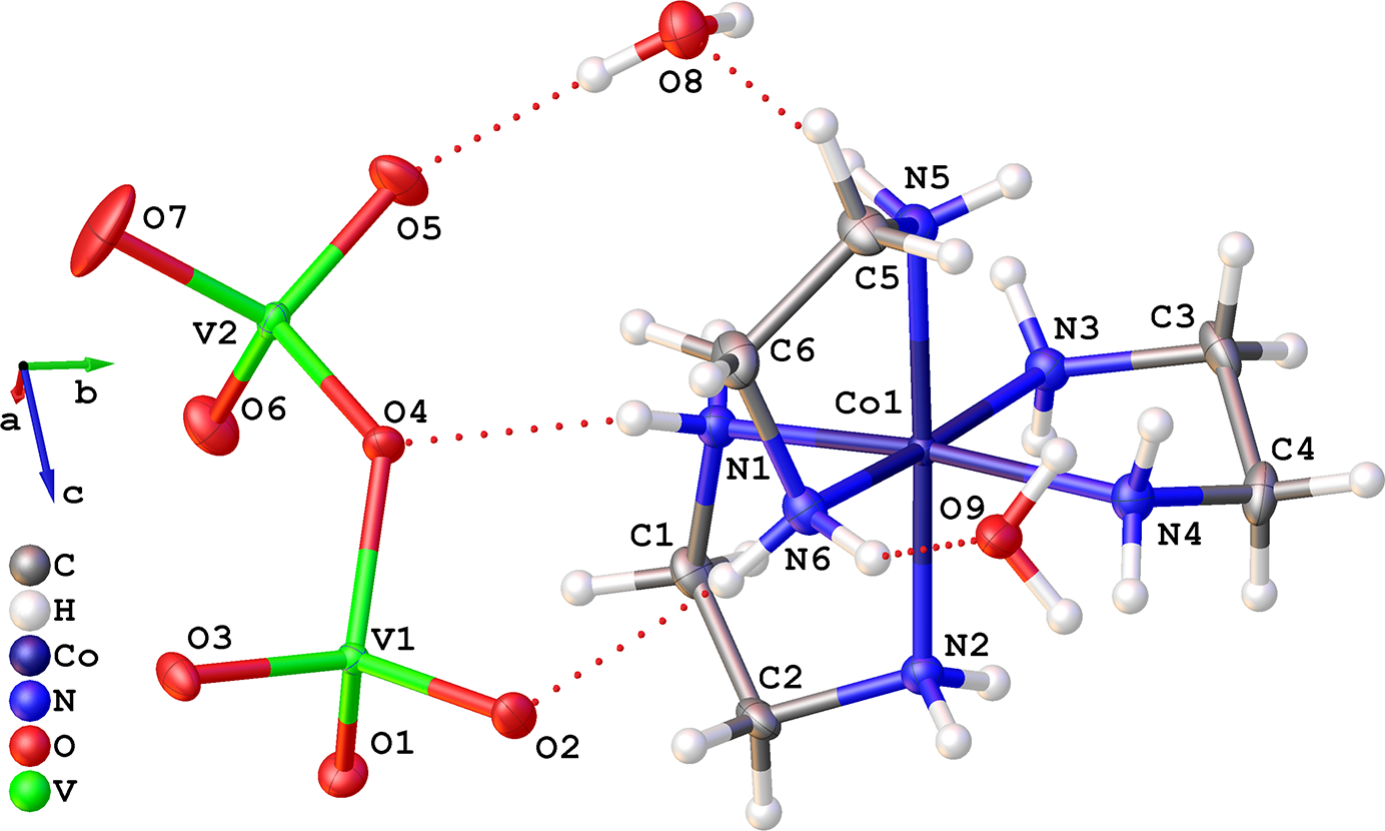
Asymmetric unit of **1**. All non-hydrogen atoms are shown as 50% probability ellipsoids. Dotted lines show hydrogen bonding.

The crystal structure of **1**, seen in Figs. 2 ▸ and 3 ▸, consists of octahedrally coordinated [Co(en)_3_]^3+^ complexes and chains of four corner sharing tetrahedral vanadate units, as well as some crystal water. The cobalt complexes and water molecules form hydrogen bonds to the vanadate chains in a comprehensive framework.

The asymmetric unit (Fig. 2 ▸) contains one full octahedrally coordinated [Co(en)_3_]^3+^ complex, half of a (V_4_O_13_)^6−^ chain, and two water molecules. All atoms are placed on general positions, except one oxygen (O7), which is placed on an inversion centre linking the two symmetry related halves of the vanadate chain. Due to the centrosymmetric space group 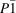 containing the inversion symmetry operation, both enantiomers of [Co(en)_3_]^3+^ are present in the crystal.

Bond lengths in the [Co(en)_3_]^3+^ complex are close to 1.5 Å for all C—C and C—N bonds with relatively low N—C—C—N torsion angles around 50°, and Co⋯N coordination distances of 1.95–1.97 Å, which are approximately 0.20 Å shorter than bond lengths reported by Pham *et al.* (2017 ▸) at 200 K. Vanadium–oxygen bond lengths are close to 1.67 Å for the partial double bonds (V1—O1/O2/O3 and V2—O5/O6) and longer, ∼1.8 Å, for the chain backbone V1—O4—V2—O7 bonds. Bond lengths reported here align well with values found in the literature (Wang *et al.*, 2004 ▸; Khan *et al.*, 2005 ▸; Liu *et al.*, 2000 ▸). The partial bond order representation of the vanadium chain shown in Fig. 1 ▸ was derived from simple resonance structure rules giving a partial V—O bond order of 1.3 for the end-unit bonds (V1—O1/O2/O3) and 1.5 for the middle-unit bonds (V2—O5/O6). This corresponds well with the bond lengths observed in the crystal structure, where the end-unit bonds are slightly longer (∼0.03 Å) than the middle-unit bonds.

**Figure 3:**
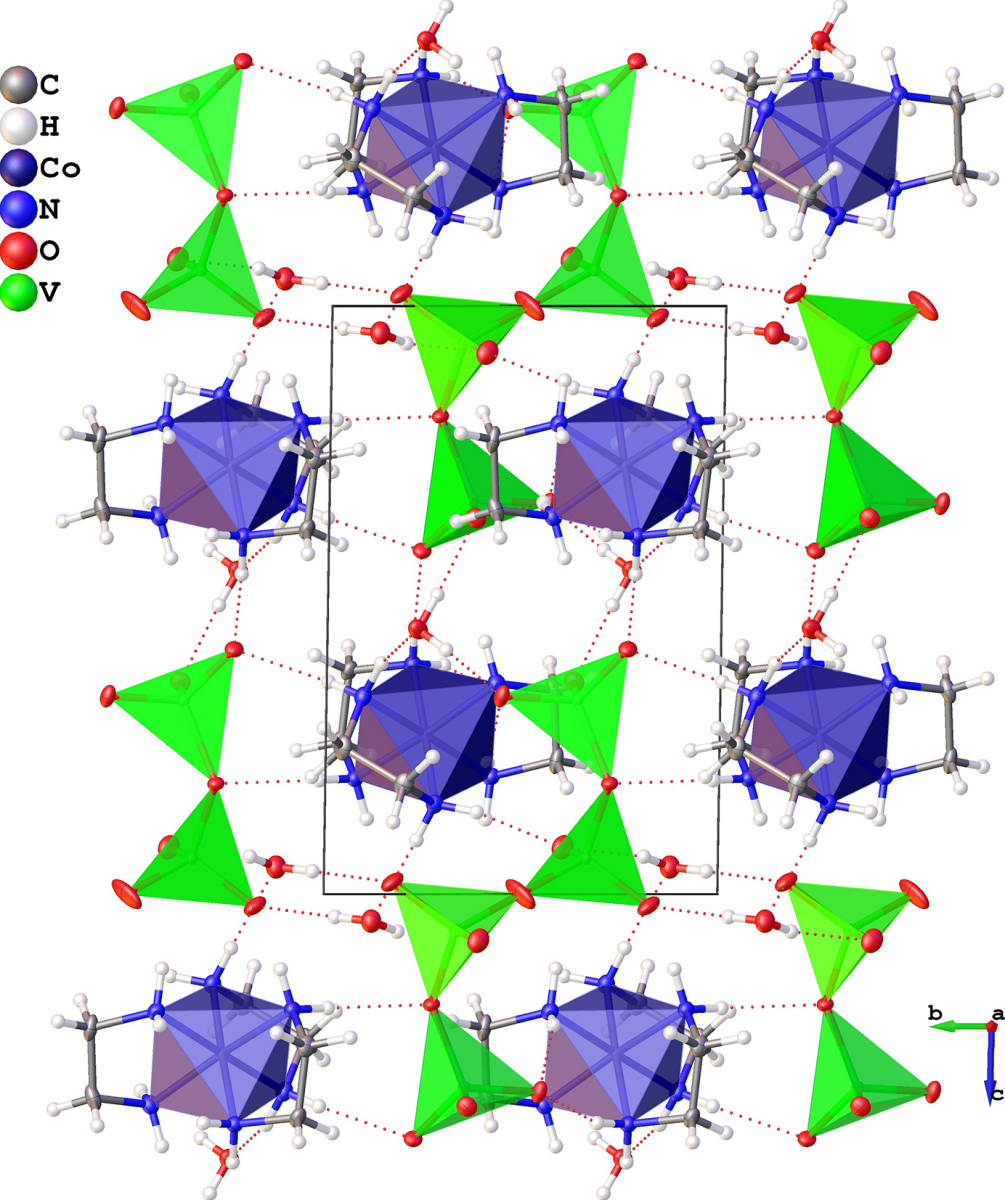
Unit cell and extended structure of **1**. All non-hydrogen atoms are shown as 50% probability ellipsoids.

The two [Co(en)_3_]^3+^ complexes and two partial vanadate chains in the unit cell (Fig. 3 ▸) form hydrogen bonds enclosing the inversion symmetry element in the centre of the cell. The vanadate chains lie in the *bc* plane along the **c** direction.

### 3.2. Extended structure and hydrogen bonding

The intermolecular interactions are governed by an extensive framework of relatively short hydrogen bonds (below 2.5 Å). All hydrogen, nitro­gen and oxygen atoms in the structure participate in hydrogen bonding with nitro­gen from en as a donor (*D*) and vanadate oxygen as an acceptor (*A*). Each water molecule donates hydrogen to a hydrogen bond with the vanadate chains and acts as an acceptor of a hydrogen bond from the [Co(en)_3_]^3+^ complex.

**Figure 4:**
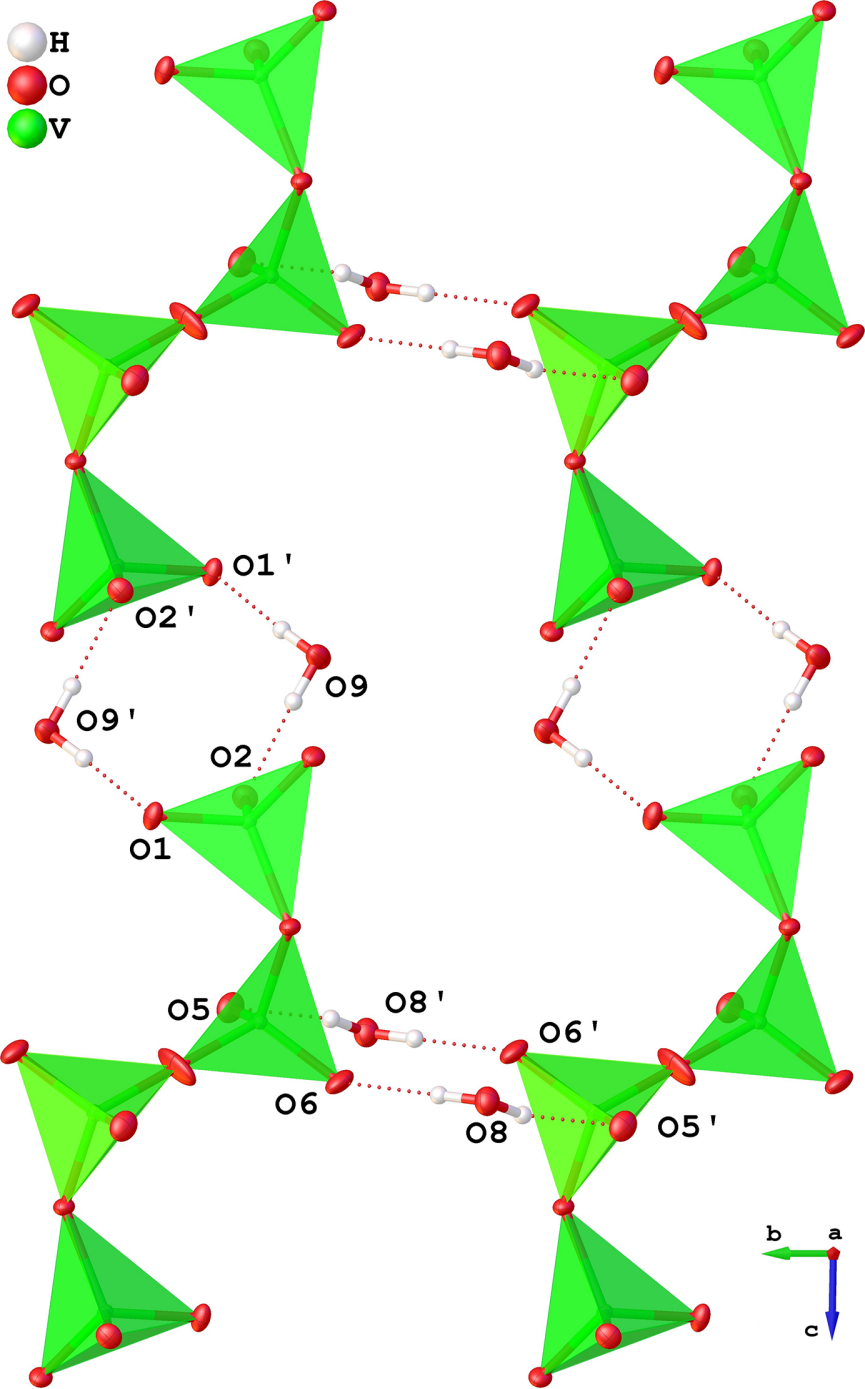
Schematics showing the extended framework of vanadate chain bridged by water molecules. The [Co(en)_3_]^3+^ complexes are omitted for clarity. Symmetry related atoms are marked by an apostrophe, the exact notation of the symmetry relation can be seen in Table 2 ▸.

The water molecules act as hydrogen bonding bridges between vanadate chains in the *bc* plane (Fig. 4 ▸). The O9 water molecule participates in hydrogen bonding with the vanadate chain end-units along the chain direction (unit cell *c* axis), while the O8 water molecule bridges in the middle-units perpendicular to the chain direction (unit-cell *b* axis). The hydrogen bonds where water acts as a donor have relatively short interaction distances of H⋯*A* < 2 Å and *D*—H⋯*A* angles of 165–176°.

In addition to the hydrogen bonds with the crystal water molecules, direct hydrogen bonding interactions between donor nitro­gen atoms in the [Co(en)_3_]^3+^ complexes and the acceptor oxygen atoms in the vanadate chains can be found. All nitro­gen atoms act as hydrogen-bond donor with the shortest being N6—H6*B*⋯O2 (see Table 2 ▸).

The interactions between the organic complex and the inorganic polyoxovanadate chain were investigated by Hirshfeld surface (HS) analysis (Spackman *et al.*, 2021 ▸), and it was found that the closest contacts are hydrogen bonds, suggesting that there are no interactions between the metals. HS analysis in Table S2 and Fig. S4 shows that the only interactions of the [Co(en)_3_]^3+^ complex are through the hydrogen atoms with either hydrogen (Fig. S4, HH) or oxygen (Fig. S4, HO) outside the surface. The closest contacts are hydrogen bonds with oxygen in the vanadate chain. Likewise the HS of the vanadium oxide chain in Fig. S5 shows that all closest contacts are through oxygen with hydrogen outside the surface. The fact that only weak interactions exist between the organic and inorganic moieties in the crystal is a characteristic feature of the hybrid crystals, but is otherwise mainly observed in organic (co-)crystals.

### 3.3. Characterization

The products of the hydro­thermal syntheses were characterized by SEM-EDX, showing peaks in the EDS spectrum for all expected elements (C, N, O, V and Co), and a homogeneous distribution of Co and V throughout the sample with a 1.2:2.5 (approx. 1:2) atomic percentage ratio of Co and V. The EDS spectra and corresponding SEM images with elemental maps can be seen in Fig. S6 and S7. It is concluded that **1** with a Co:V ratio of 1:2 is the exclusive product of the syntheses for all tested molar ratios of the precursors, CoCl_2_·6H_2_O and NH_4_VO_3_.

FT–IR data was measured on a powder sample of the synthesis product and shows peaks consistent with N—H stretching from an amine group and O—H stretching from water in the region 3300–3000 cm^−1^, as well as the corresponding N—H and O—H bending peaks around 1575 cm^−1^. Absorption bands associated with C—H stretching are found in the region 3000–2900 cm^−1^, consistent with other reports on the ethyl­enedi­amine ligand (Baldwin, 1960 ▸; Righini & Califano, 1976 ▸). One sharp band around 1000 cm^−1^ is assigned to V—O stretching (Frederickson & Hausen, 1963 ▸). Characteristic absorption bands in the fingerprint region around 600 cm^−1^ are characteristic for metal–N coordination (Hughes & McWhinnie, 1966 ▸). The FT–IR spectrum can be seen in Fig. S8. It should also be noted that the compound is stable at ambient conditions. Comparison of IR spectra of the freshly synthesized samples and samples after seven months storage showed that they were the same, *i.e.* no changes were observed (Fig. S9).

High-quality synchrotron PXRD patterns at several temperatures were obtained from beamline BL44B2 at Spring-8, Japan. The PXRD data were modelled with the structure obtained from SCXRD, and fits are shown in Figs. S10–S11 and further discussed in the supporting information. No impurity phases were identified (Figs. S12–S13), and all Bragg reflections were described by the single-crystal structure No phase transition was observed upon cooling, or subsequent reheating. Intensity deviations are ascribed to poor powder statistics or disorder in the crystal structure, as also observed with SCXRD (see discussion in supporting information). The results from PXRD confirm that the sample is phase pure and that **1** is the exclusive product of the synthesis.

### 3.4. Properties

#### 3.4.1. Thermal expansion

**Figure 5:**
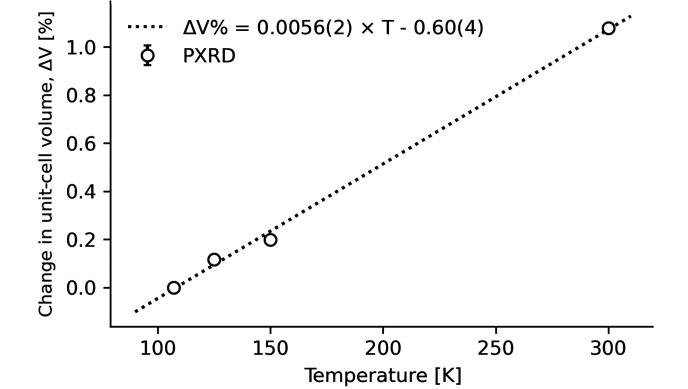
Relative thermal expansion of the unit-cell volume of **1**. The percentage change in unit-cell volume is found as: Δ*V*% = (*V* − *V*_0_)/*V*_0_ × 100%, where *V*_0_ is the volume at 107 K. The dotted line shows the linear regression expressed as shown in the figure.

Materials with a large thermal expansion can be impractical for implementation in potential applications. A large thermal expansion coefficient can arise from weak bonding as observed in the organic–inorganic hybrid crystals (Ge *et al.*, 2018 ▸). Unit-cell volume expansion of **1** as a function of temperature obtained by PXRD is shown in Fig. 5 ▸. The volume thermal expansion coefficient was found by linear regression to be 56 (2) × 10^−6^ K^−1^ with a unit-cell expansion of about 1% in the temperature range from 100 to 300 K. Changes in unit-cell-edge lengths (*a*, *b* and *c*) follow similar curves with expansions between 0.2% and 0.8% in the reported temperature range, while the unit-cell angles (α, β and γ) decrease slightly with reductions of −0.1% to −0.3% (Table S3 and Fig. S14). The linear thermal expansion coefficient is as high as 43 (2) × 10^−6^ K^−1^ along the [100] direction.

#### 3.4.2. Electronic properties

**Figure 6:**
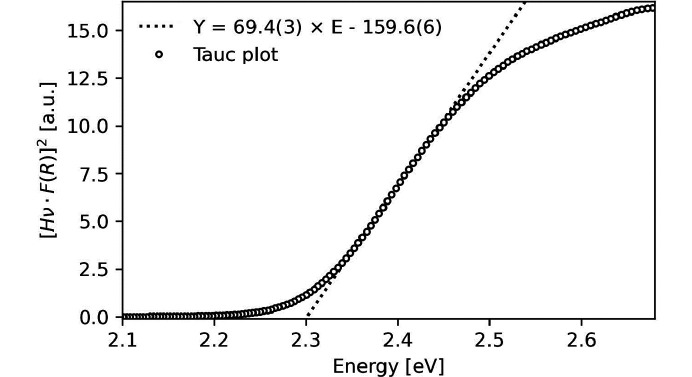
Direct band gap energy of **1** obtained from UV–vis. The dotted line shows the linear regression expressed as shown in the figure, *Y* = [*H*ν · *F*(*R*)]^2^, where *H*ν is the energy in eV and *R* is the reflectance.

Organic–inorganic hybrid crystals, especially the perovskites, have been extensively studied due to their photovoltaic performance. For practical relevance, this requires a band gap around 1.1 eV and a high power conversion efficiency for a single material, but advances have been made for, for example, multi-junction solar cell *etc*. using also higher band gap materials (Ajayan *et al.*, 2020 ▸). The band gap of **1** was determined by UV–vis spectroscopy to be 2.30 (1) eV. Fig. 6 ▸ shows the Tauc plot used to estimate the band gap.

#### 3.4.3. Magnetic properties

**Figure 7:**
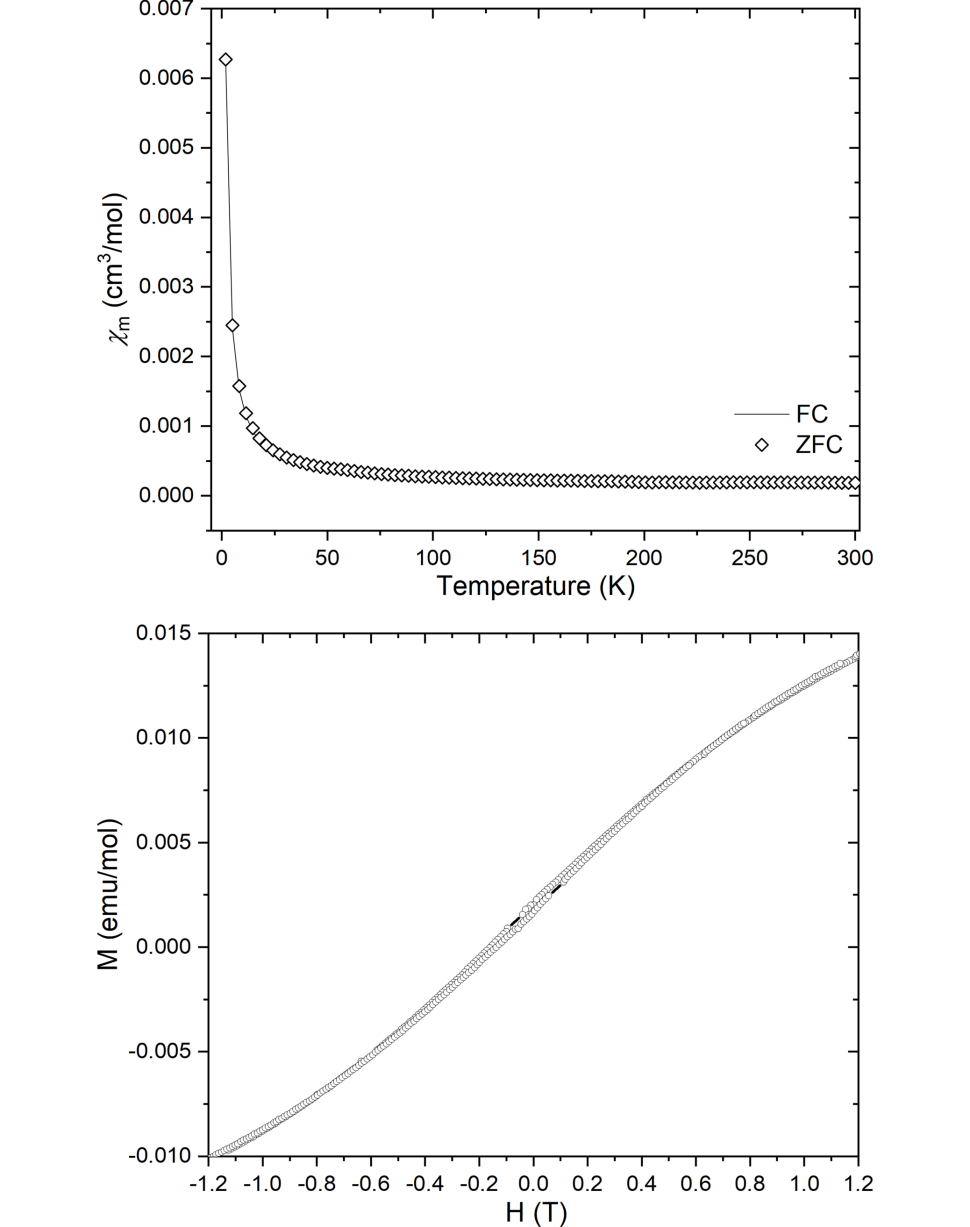
(Top) Temperature dependence of the molar magnetic susceptibility for **1** under FC and ZFC mode at 500 Oe external field. (Bottom) Magnetic hysteresis measure for **1** at 1.8 K from −1.2 to 1.2 T.

Vanadium in the +V oxidation state has *d*^0^ electron configuration and does not contribute to the magnetic properties (Liu *et al.*, 2000 ▸). Cobalt in the +III oxidation state has *d*^6^ electron configuration, and usually only persists as low spin with no unpaired electrons in a perfect octahedral field. Based on these theoretical considerations the sample should behave as a diamagnet. Static (DC) magnetometry was performed on **1** to gauge its magnetic response. Temperature dependences of the molar magnetic susceptibility of **1** was studied under ZFC and FC modes under 500 Oe external field (Fig. 7 ▸). The FC and ZFC curves overlap indicating no abrupt magnetic ordering transition. The molar magnetic susceptibility (χ_m_) value at 300 K was around 1.8 × 10^−4^ cm^3^ mol^−1^ (corresponding to around 0.67 BM) and remains steadily low until around 50 K after which it starts to increase steeply at lower temperatures reaching 6.3 × 10^−3^ (≈0.30 BM) at 1.8 K. This indicates low-spin Co^III^ behaviour at room temperature with contributions from small Co^II^ impurities at the Co^III^ site giving rise to mild paramagnetic behaviour at low temperatures. This is consistent with results from PXRD showing no presence of additional crystalline phases. Furthermore, a magnetic hysteresis loop was recorded at 1.8 K showing no significant opening around zero external field. Non-linear variation of magnetization was observed at higher fields and low temperatures. Results presented here are consistent with reports from Kahn *et al.* (2005 ▸). Calculations to quantify the amount of Co^II^ impurity based on the magnetic response have been performed, suggesting ∼0.7% Co^II^ impurity in the sample (see supporting information).

## 4. Conclusions

In this work the structure and properties of a new mixed metal CoV compound, **1**, 2[Co(en)_3_]^3+^(V_4_O_13_)^6−^·4H_2_O was reported. The organic–inorganic hybrid crystal structure solved by SCXRD consists of [Co(en)_3_]^3+^ octahedra and (V_4_O_13_)^6−^ chains with four corner-sharing tetrahedral VO_4_ units. Characterization by SEM-EDX and FT–IR showed that **1** is the sole product of this very robust novel hydro­thermal synthesis when the molar ratios of the precursors CoCl_2_·6H_2_O and NH_4_VO_3_ were varied between 1:3, 1:2, 1:1 and 2:1. Phase identification by PXRD supported this conclusion. The volume thermal expansion coefficient was found to 56 (2) × 10^−6^ K^−1^ with a unit-cell expansion of 1% in the reported temperature range of 100–300 K. The band gap of **1** was found by UV–vis to be 2.30 (1) eV and magnetic susceptibility measurements down to 1.8 K showed that the compound exhibits a weak paramagnetic response at low temperatures, attributed to minor Co^II^ impurities at the Co^III^ site.

## Supplementary Material

Crystal structure: contains datablock(s) I. DOI: 10.1107/S2052520624007509/lo5123sup1.cif

Structure factors: contains datablock(s) I. DOI: 10.1107/S2052520624007509/lo5123Isup2.hkl

Includes Tables S1-S3 and Figs. S1-S14. DOI: 10.1107/S2052520624007509/lo5123sup3.pdf

CCDC reference: 2311892

## Figures and Tables

**Table 1:** Crystallographic information for compound **1**

Crystal data	
Asymmetric unit	[Co(en)_3_](V_2_O_6.5_)·2(H_2_O)
*M*_r_	481.15
Crystal system, space group	Triclinic, *P* 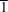
Temperature (K)	150 (2)
*a*, *b*, *c* (Å)	8.6226 (3), 8.9645 (3), 12.8206 (5)
α, β, γ (°)	81.491 (3), 71.357 (3), 65.064 (4)
*V* (Å^3^)	851.36 (6)
*Z*	2
Radiation type	Mo *K*α
μ (mm^−1^)	2.09
F(000)	494
ρ (g cm^−3^)	1.877
Crystal size (mm)	0.09 × 0.08 × 0.03

Data collection	
Diffractometer	XtaLAB Synergy, Dualflex, HyPix-Arc 100°
Absorption correction	Multi-scan (*CrysAlis PRO*). Empirical absorption correction using spherical harmonics, implemented in SCALE3 ABSPACK scaling algorithm.
*T*_min_, *T*_max_	0.910, 1.000
No. of measured, independent and observed [*I* > 2σ(*I*)] reflections	26667, 5099, 4778
*R*_int_	0.018
(sin θ/λ)_max_ (Å^−1^)	0.746

Refinement	
*R*[*F*^2^ > 2σ(*F*^2^)], *wR*(*F*^2^), *S*	0.027, 0.073, 1.06
No. of reflections	5099
No. of parameters	221
H-atom treatment	H atoms treated by a mixture of independent and constrained refinement
Δρ_max_, Δρ_min_ (e Å^−3^)	1.30, −0.47

**Table 2:** Hydrogen-bond geometry (Å, °)

*D*—H⋯*A*	*D*—H	H⋯*A*	*D*⋯*A*	*D*—H⋯*A*
O8—H8*A*⋯O5^i^	0.87	1.990	2.833 (3)	164.7 (1)
O8—H8*B*⋯O6	0.87	1.896	2.763 (2)	174.4 (1)
O9—H9*A*⋯O2	0.87	1.858	2.727 (3)	176.1 (1)
O9—H9*B*⋯O1^ii^	0.87	1.905	2.769 (2)	171.4 (1)
N6—H6*B*⋯O2	0.910	1.987	2.730 (2)	137.7 (1)

Symmetry codes: (i) 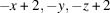; (ii) 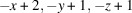.
