# Supplementary material for: Synthesis and characterization of an organic–inorganic hybrid crystal: 2[Co(en)3](V4O13)·4H2O
Source: Acta Crystallogr B Struct Sci Cryst Eng Mater. 2024 Sep 3;80(Pt 5):488–94. doi: 10.1107/S2052520624007509 (PMC11457104; doi:10.1107/S2052520624007509)

STRUCTURAL SCIENCE
CRYSTAL ENGINEERING
MATERIALS

Volume 80 (2024)

Supporting information for article:

Synthesis and characterization of an organic–inorganic hybrid crystal: $2[Co(en)_3](V_4O_{13})\cdot 4H_2O$

Emilie Skytte Vosegaard, Mohammad Aref Hasen Mamakhel, Vijay Singh Parmar, Andreas Dueholm Bertelsen and Bo Brummerstedt Iversen

S1. Synthesis

Four different syntheses were prepared with different ratios (1:3, 1:2, 1:1 and 2:1) of cobalt and vanadium precursors. All synthesis routes gave the same crystalline products of brittle plate-like transparent golden single crystals as seen in Figure S1. The following section shows the results from single crystal experiments measured on all four products, as well as presenting the difficulties in data treatment and structure solution.

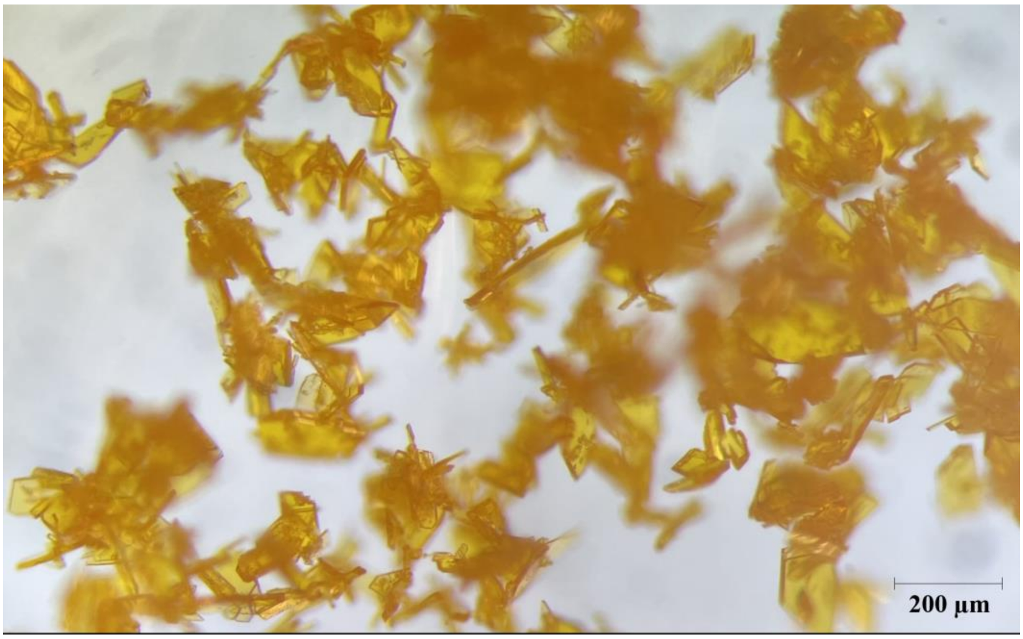

Figure S1 Image of the single crystals of **1** by visible microscope. The scale bar is 200 µm.

S2. Crystallographic experiments

Samples are named according to the Co:V ratio in the synthesis, as: CoV-1-3, CoV-1-2, CoV-1-1 and CoV-2-1. The structure presented in the main manuscript is here mentioned as sample CoV. All batches showed to give the same crystalline products as presented in Table S1. Attempts were made to obtain low temperature datasets for better structure determination, but the crystals deteriorated during the prolonged cooling needed for a sufficient dataset. Datasets obtained at 100 K for CoV-1-2 and CoV-2-1 showed intrinsic twinning and disorder, respectively. The room temperature datasets obtained from the CoV-1-3 and CoV-1-1 crystals performing slightly better than the other datasets in terms of quality parameters, as reported in Table S1. Figure S2 shows the CoV crystal used for the experiment.

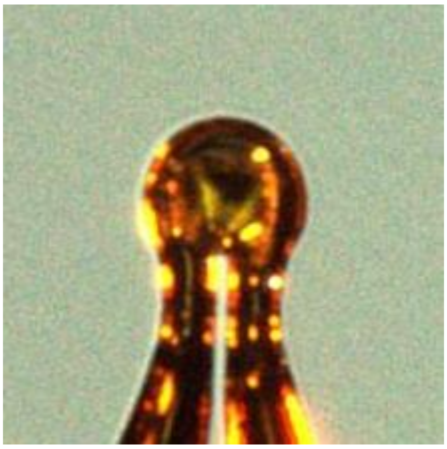

Figure S2 The crystal used for the single crystal X-ray diffraction experiment presented in the paper. The hoop has a diameter of 100 µm.

Table S1 Crystallographic information.

Crystal data	CoV-1-3	CoV-1-2	CoV-1-1	CoV-2-1
Chemical formula	$[Co(en)_3](V_2O_{6.5})\cdot 2(H_2O)$	$[Co(en)_3](V_2O_{6.5})\cdot 2(H_2O)$	$[Co(en)_3](V_2O_{6.5})\cdot 2(H_2O)$	$[Co(en)_3](V_2O_{6.5})\cdot 2(H_2O)$
Formula weight	481.15	481.15	481.15	481.15
Crystal system, space group	Triclinic, *P*-1	Triclinic, *P*-1	Triclinic, *P*-1	Triclinic, *P*-1
Temperature	RT	100 K	RT	100 K
a, *b*, *c* (Å)	8.6659(6), 8.9750(6), 12.7974(9)	8.6134(2), 8.9591(3), 12.8049(4)	8.6822(7), 8.9764(7), 12.7949(11)	8.6053(3), 8.9571(4), 12.8067(4)
α, β, γ (°)	81.436(6), 71.208(6), 64.909(7)	81.598(3), 71.468(3), 65.051(3)	81.502(7), 71.199(7), 64.849(8)	81.620(3), 71.499(3), 65.094(4)
V (Å^3)	853.27(12)	849.38(5)	854.40(14)	848.96(6)
Z	2	2	2	2
Radiation type	Mo *K*α	Mo *K*α	Mo *K*α	Mo *K*α
µ (mm^{-1})	2.03	2.03	2.03	2.03
F(000)	494.0	494.0	494.0	494.0
ρ (g cm^{-3})	1.87	1.87	1.87	1.87

Crystal size (mm)	0.16 × 0.09 × 0.04	0.21 × 0.13 × 0.05	0.23 × 0.11 × 0.08	0.06 × 0.05 × 0.02
R_{int}	0.058	0.052	0.049	0.037
Completeness (%)	99.9	100	100	99.9
d_{min} (Å), $(\sin \theta/\lambda)_{max}$ (Å^{-1})	0.83, 0.602	0.70, 0.714	0.80, 0.625	0.70, 0.714
Collected, unique reflns.	10919, 10233	8591, 8591	19095, 14087	19886, 19886
$R(F^2)$, $wR(F^2)$, *GOF*	0.038, 0.080, 1.016	0.043, 0.123, 1.085	0.036, 0.093, 1.03	0.039, 0.103, 1.035
$\Delta\rho_{max}$, $\Delta\rho_{min}$ (e Å^{-3})	0.5, -0.3	1.8, -0.6	0.86, −0.35	1.7, -0.5
Comment	OK	Twin	OK	Disorder

CoV-1-3: Measured at room temperature to avoid crystal scattering during cooling. Powder rings, probably from the sample mount, observed on some frames.

CoV-1-2: Visual inspection of the frames show that ice crystals formed during the measurement, so only the two first runs were used for the unit cell finding. The frames suggested twinning, confirmed by lower R factors, when two twin components are used for the model. The integration refines to a 54/46 ratio of the two components. All frames were used in the final integration.

CoV-1-1: Previous attempts to find a good crystal failed due to the brittle nature of the crystals. Like the others, this one forms needle shaped crystals that agglomerates and easily breaks. A lot of what appears to be single crystals turn out to be multi crystals (further attempts to solve these structures were unsuccessful, due to >2 twin domains) or intrinsical twinning (like CoV-1-2). Upon cooling to 100 K some of the crystals broke, so the reported structure was obtained at RT.

CoV-2-1: Residuals in an octahedral shape around Co suggest some disorder in the ligands. Keeping the same Co-center, but allowing the ethylenediamine units to split, refines to a 92/8 ratio of the two conformations and lowers the R-factor from 3.88 to 2.87. The max residual is decreased as well (from +1.7 e/Å^3 to +0.7 e/Å^3), but the ADPs of the 8% fraction of the $Co(en)_3$ complex have unphysical values when refined both isotropically and anisotropically as seen in Figure S6. Since the disorder only has a minor contribution to the final model, as evident from the already low value of the max/min residuals and the low R-factor, it is not included in the final model.

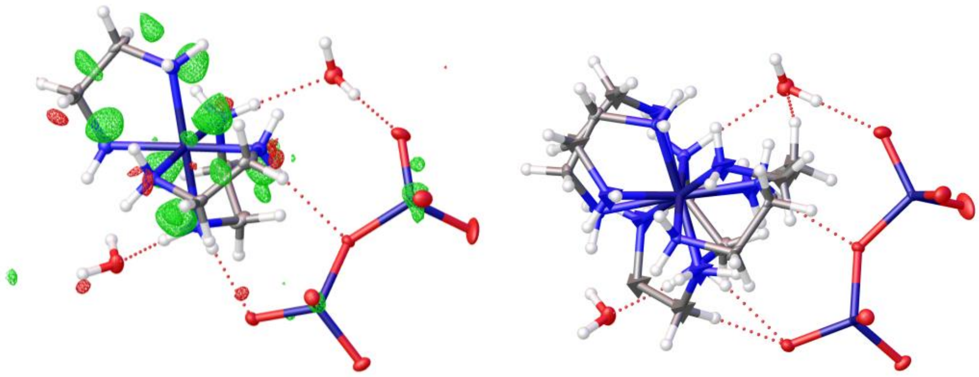

Figure S3 Residuals in the CoV-2-1 structure, and the disordered solution with unphysical ADPs.

S3. Hirshfeld Surface analysis

Results from the Hirshfeld Surface (HS) analysis can be seen in Table S2, Figure S4 and Figure S5.

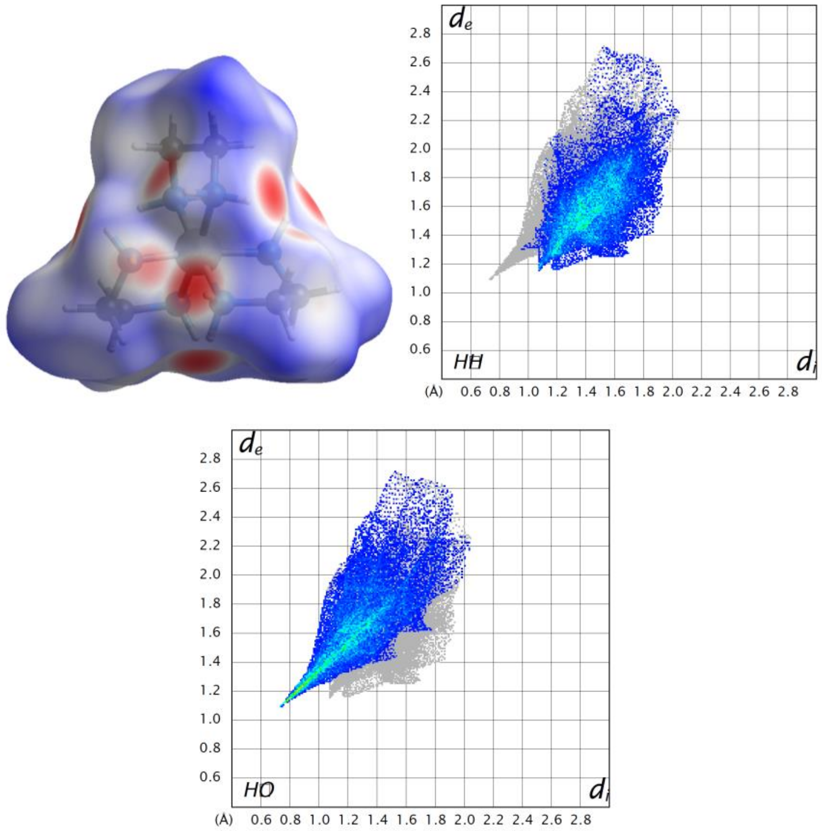

Figure S4 Hirshfeld Surface analysis of the $[Co(en)_3]$ complex showing the HS and fingerprint plots (d_e, distance from nearest external atom to HS, vs d_i, distance from nearest internal atom to HS) of the H_{inside}-$H_{outside}$ and H_{inside}-$O_{outside}$ contacts.

Table S2 Hirshfeld Surface analysis. 100% of the contacts from the [Co(en)$_3$] cluster are from hydrogen atoms inside the surface.

Atom outside	Surface area %
Co, N and C	0
V	0.4
O	48.2
H	51.4

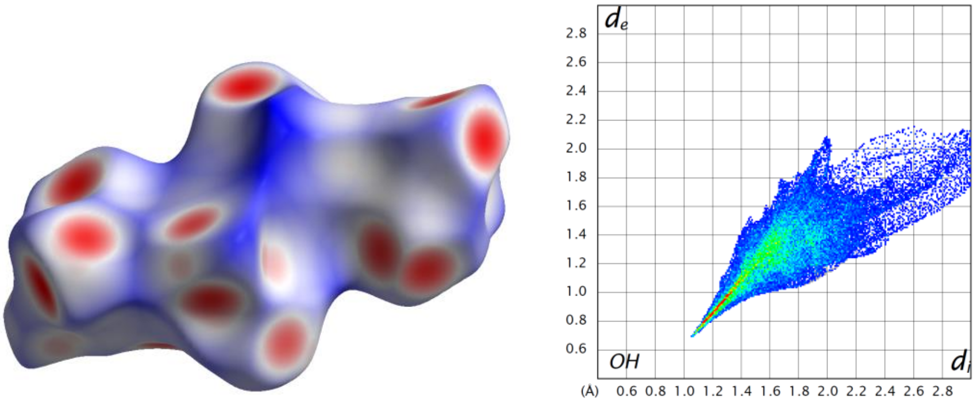

Figure S5 Hirshfeld Surface analysis of the (V_4O_{13}) chain showing the HS and fingerprint plot (d_e, distance from nearest external atom to HS, vs d_i, distance from nearest internal atom to HS) of the O_{inside}-$H_{outside}$ contacts.

S4. Characterization: SEM-EDX

The product of the synthesis was characterized by SEM-EDX as seen in Figure S6 and S7.

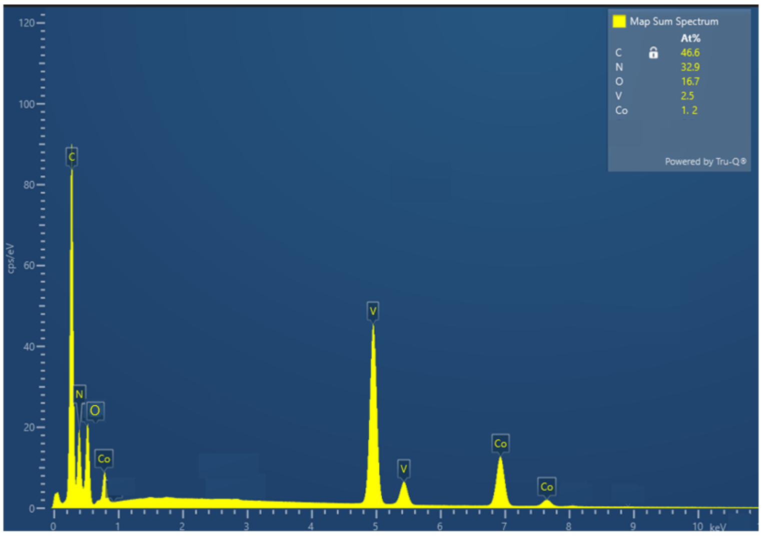

Figure S6 EDS spectrum of **1** crystals by SEM.

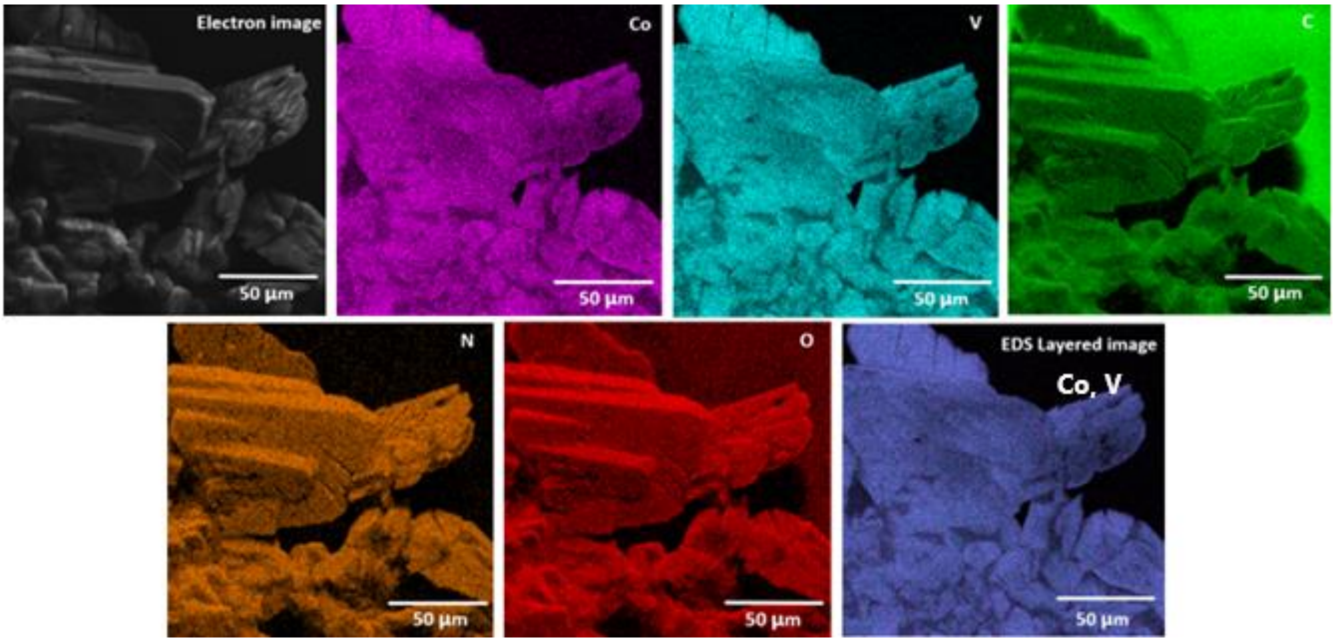

Figure S7 Electron image of **1** crystals by SEM and EDS elemental maps if Co, V, C, N, O and a superposition of Co and V.

S5. Characterization: FT-IR

In the FT-IR spectrum of **1** in Figure S8 we assume that bands >3000 cm^{-1} assign to N-H (amine stretching) and O-H (water stretching), while C-H related absorption bands are seen just below 3000

cm^{-1}. Absorption peaks at 1580 cm^{-1}, 1460 cm^{-1}, 1055 cm^{-1} and 810 cm^{-1} are characteristic for O-H (water bending), C-H, C-N and N-H (amine bending), respectively. The sharp band just below 1000 cm^{-1} is characteristic for the V-O stretching mode of the terminal V-O group. The intense peaks around 500-700 cm^{-1} are characteristic for metal-N bonds.

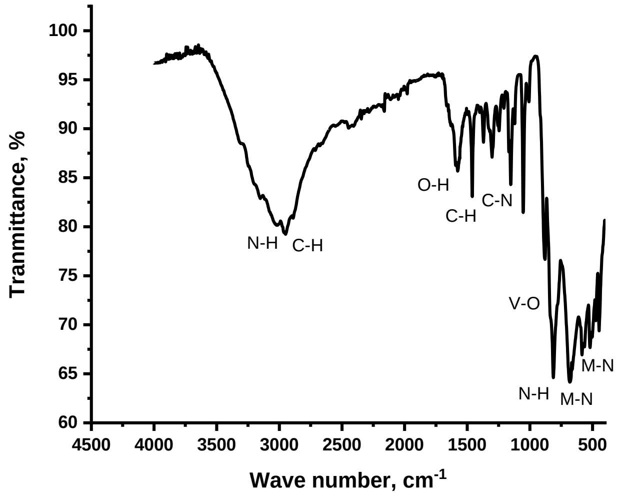

Figure S8 FT-IR spectrum of **1**.

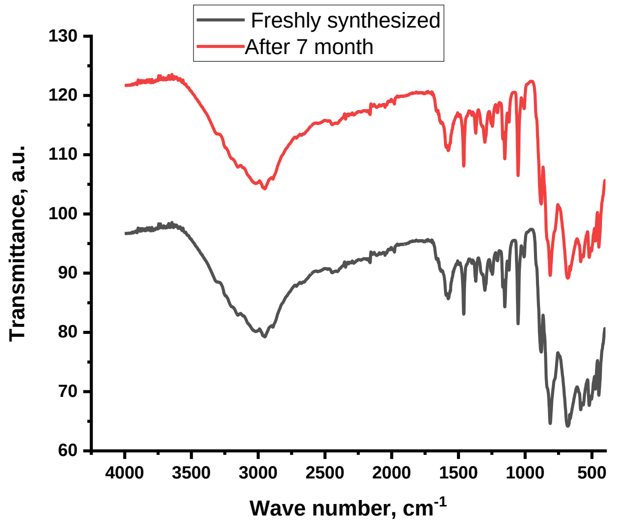

Figure S9 FT-IR spectrum of the freshly synthesized sample of **1**, and after seven months.

S6. Characterization: PXRD

The diffraction patterns of **1** measured at 107 K and 300 K are shown in Figure S10 and S11. Obtained agreement factors and refined parameters are shown in Table S3.

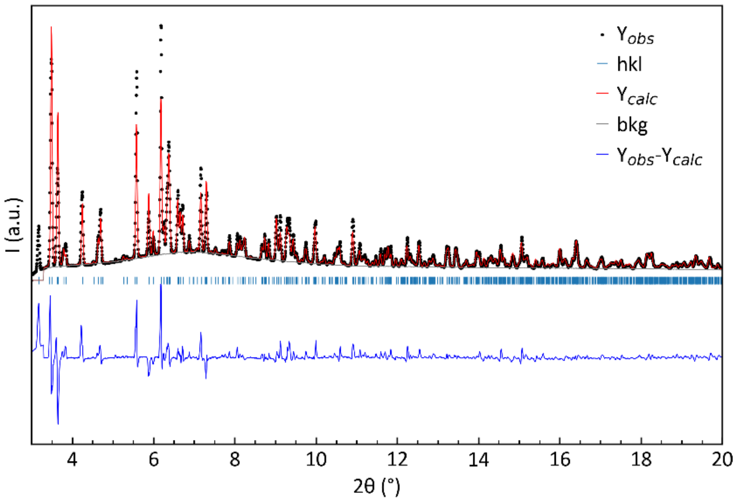

Figure S10 The observed diffraction pattern of **1** at 107 K (black), the modelled pattern (red), and background (grey). The residual is shown in blue. Obtained agreement factors are R_{wp} = 11.32 % and R_{bragg} = 7.40 %.

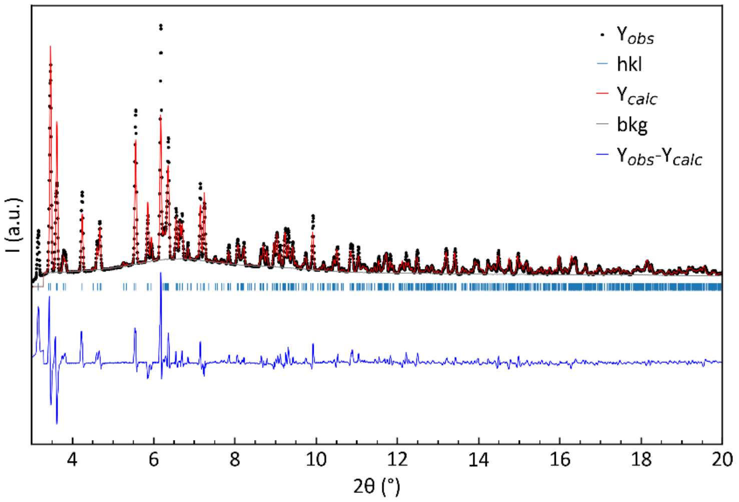

Figure S11 The observed diffraction pattern of **1** at 300 K (black), the modelled pattern (red), and background (grey). The residual is shown in blue. R_{wp} = 11.58 % and R_{bragg} = 7.74 %.

PXRD is used for phase identification, to test purity of the sample used for magnetic measurements and to analyse thermal expansion. The most probable impurity phases are the metal oxides of cobalt and vanadium, but comparison of the position of the Bragg peaks shows no significant correlation with the

observed diffraction pattern. In Figure S12 and S13 the observed diffraction pattern and the residual curve, respectively, are compared to known oxide phases of cobalt and vanadium.

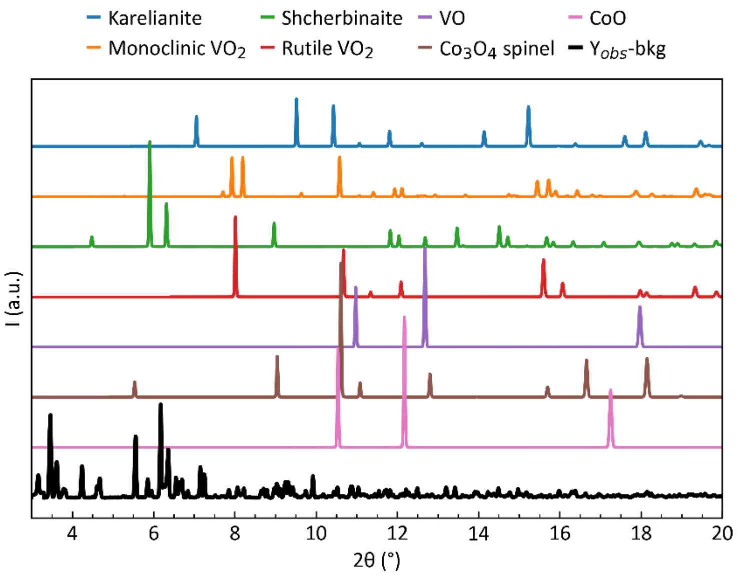

Figure S12 The observed diffraction pattern (minus the back ground, Yobs-bk) of **1** at 300 K (black), compared to possible oxide phases of cobalt and vanadium.

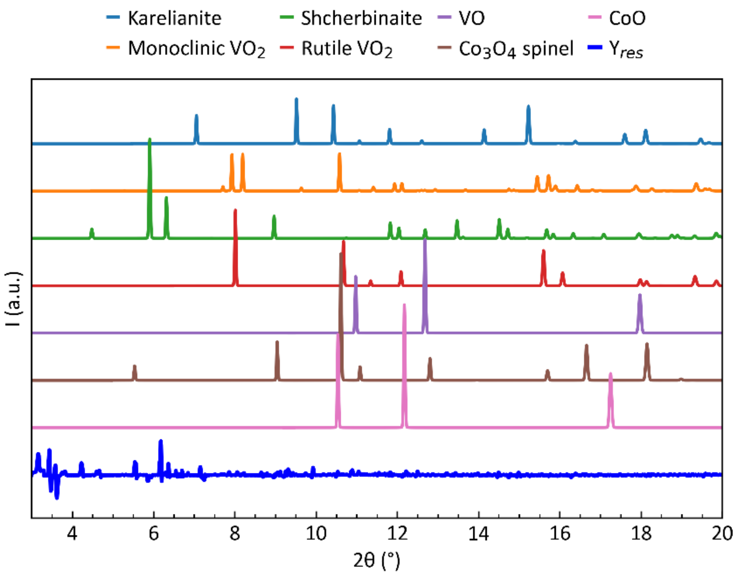

Figure S13 The residual curve of the fit (Yobs-Ycalc) of **1** at 300 K (blue), compared to possible oxide phases of cobalt and vanadium.

Table S3 Refined unit cell parameters for PXRD of **1**.

Temperature (K)	107	125	150	300
a, *b*, *c* (Å)	8.6289(3), 8.9702(3), 12.8116(5)	8.6344(3), 8.9729(3), 12.8169(5)	8.6390(3), 8.9751(3), 12.8193(5)	8.6993(3), 9.0024(3), 12.8380(5)
α, β, γ (°)	81.537(4), 71.445(3), 65.102(3)	81.529(3), 71.428(3), 65.093(3)	81.528(3), 71.414(3), 65.083(3)	81.431(4), 71.194(3), 64.908(3)

Relative percentage change in unit cell parameters found by PXRD as a function of temperature is shown in Figure S14. Unit cell parameters for the 107 K and 300K structures are reported in Table S3. The percentage difference is calculated as: $\Delta V\% = \frac{V - V_0}{V_0} \cdot 100\%$, where V_0 is the volume at 107 K. Other unit cell parameter differences are calculated correspondingly. The linear expansion coefficienta of in the *a*, *b* and *c* directions are 43(2) · 10^{-6} K^{-1}, 19(1) · 10^{-6} K^{-1} and 10(1) · 10^{-6} K^{-1}, respectively.

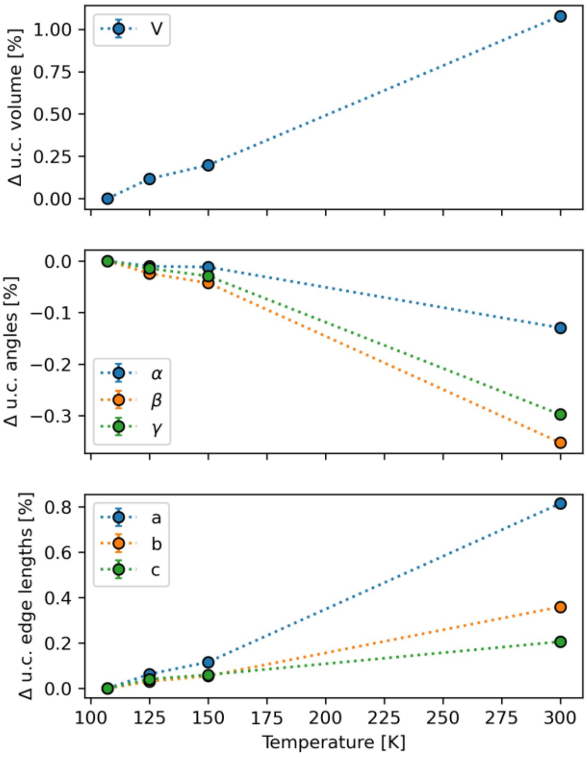

Figure S14 Relative change in unit cell parameters obtained from PXRD as a function of temperature.

S7. Quantification of impurity fraction:

To account for the trace impurity giving rise to the paramagnetic behavior, the molar magnetic susceptibility data was fitted with eq:

$$\chi = (1 - IMP)\frac{\chi_{calc} + TIP}{1 - \left(\frac{zJ}{N_A{\mu_B}^2}\right)(\chi_{calc} + TIP)} + (IMP)\chi_{IMP}$$

Where, TIP, IMP, X_IMP and Zj represent the temperature independent paramagnetism, fraction of the impurity, susceptibility from the impurity and intermolecular interaction parameter respectively. The resultant parameters are TIP = 0.15 ± 1.28 cm3 mol^{-1} , IMP = 0.0067 ± 6.7x10^{-5} and Zj = -0.18 ± 0.15 with residual = 6.7 x 10^{-5}, indicating ~0.7% Co(II) impurity in the sample. Using this percentage impurity M vs H data was calculated (fig, red line) which seems reasonable given the model does not account for the background magnetization and any spin-orbital coupling contribution in the Co(II) impurity.

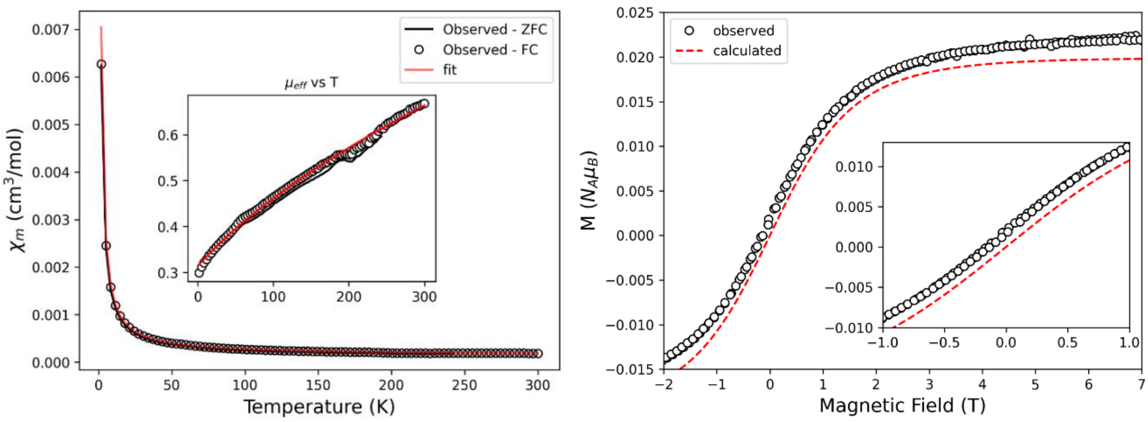

Supplement: Supplementary file 3 [file b-80-00488-sup3.pdf]
